# Are Metropolitan Areas Primed for Success? A Prosperity Risk Index for Evaluating Economic Development Patterns

**DOI:** 10.17645/up.v5i3.3151

**Published:** 2020-09-29

**Authors:** Richard Sadler, Dayne Walling, Zac Buchalski, Alan Harris

**Affiliations:** 1 Department of Family Medicine, Michigan State University, Flint, MI 48502, USA;; 2 Division of Public Health, Michigan State University, Flint, MI 48502, USA;; 3 Department of Geography, Environment, and Society, University of Minnesota, Minneapolis, MN 55455, USA;

**Keywords:** economic development, inequality, land use planning, prosperity, regional studies, spatial analysis, urban geography

## Abstract

Urban areas differ greatly in their exposure to economic change, their trajectory toward recovery and growth, and the extent to which development and equity are paired. Some of this differentiation can be explained by regional dynamics, policies, and migration flows that influence the composition of economic activity, land use, and population characteristics. Simultaneously, the fortunes of center cities are known to often correlate with metropolitan characteristics, yet the interaction of socio-spatial conditions with multi-level governance and development processes—particularly with respect to how prosperity is shared across municipal lines and is distributed among communities—is under-researched. In this article, we use a GIS-based and quantitative approach to characterize such patterns and evaluate regional differences among 117 mid-sized metropolitan areas in the Eastern US with a population between 250,000 and 2,500,000. Our analysis rests on initial GIS-based inquiries to define city, urbanized area, county, and core-based statistical area-level measures of municipal fragmentation, geographic sprawl, racial segregation, economic inequality, and overall poverty. These five characteristics are combined to propose a prosperity risk index for each region. Further, indicators of economic performance such as job and population growth are inverted to create an economic vulnerability index. An interaction model is run to determine relationships among the indices to highlight both the regional differences in these characteristics that became noticeably significant in the analysis and the linkages of spatial patterns of economic growth and social equity. Analyzing these multi-scalar regional dynamics illuminates the socio-spatial patterns that deserve attention in urban economic development theory and, subsequently, offers a framework for evaluating public policy and development practices. We likewise offer two comparisons of outliers as a means of illustrating potential directions urban areas can take toward economic development. These findings are valuable for local economic development practitioners who may be seeking further contextual/comparative information on urban regions, or for others interested in understanding the dynamics behind urban planning that may drive regional competitiveness and prosperity.

## 1. Introduction

The persistent struggles of distressed central cities, particularly older industrial cities and those with large communities of color, represent significant unresolved challenges to contemporary urban planning and local economic development policy and practice. Analyses of population or economic change alone are insufficient for understanding the complex interaction of, for instance, the multiple factors contributing to concentrations of poverty, residential segregation, and variations in health outcomes (Sadler & Lafreniere, 2017; Squires & Kubrin, 2005). To respond to these issues, the spatial relationships between characteristics of development and equity need to be identified and described at the scales commensurate with the levels of public and other governance institutions that set and put into practice local economic development policies.

Our main starting points are: 1) the importance of the wider metropolitan context in affecting fortunes of central cities (Hill, Wolman, Kowalczyk, & St. Clair, 2012; Wolman, Hill, Blumenthal, & Furdell, 2008); 2) the recognition that “the real city is the total metropolitan area—city and suburb” (Rusk, 1993, p. 5); and 3) the significance of the linkage of social equity with sustained economic growth (Benner, 2015; Pastor & Benner, 2008). Practically, how do US metropolitan areas in different regions compare in terms of economic performance and prosperity? Furthermore, what are the barriers and opportunities around equitable economic development of applicable state, metropolitan, county, and city levels? Taking a problem-driven approach and analyzing the multi-scalar metropolitan dynamics is intended to be useful for policymakers and practitioners aiming to improve their municipalities equitably (Markusen, 2015). These considerations are important for the fields of local planning and economic development, since they bring attention to what may be overlooked in many inquiries on the subject and they have implications for quality of life.

### 1.1. Comparative Work on Regional Economic Development

One major challenge in the study of urban development is the difficulty of taking into account the varied geographic elements of space, place, and scale. In fact, the scholarly work on urban redevelopment representing a wide range of academic disciplines often lacks an attention to scale. For instance, a significant body of work has examined the development trajectories of shrinking or older industrial places in the US (Beauregard, 2012; Booth, 1986; Hill, Wolman et al., 2012) and established that the statistical measures of industry sector and firm mix, workforce skills and human capital, and sub-national regional demographic forces (i.e., the growth of the Sunbelt) have explanatory power with differing development patterns. A portion of the phenomenon of uneven redevelopment, however, remains undetermined according to recent assessments by the Federal Reserve Bank (Kodrzycki & Muñoz, 2015). Data in a recent report from the Brookings Institute shows that more than half of the urban counties that have lost the greatest shares of manufacturing employment since 1970 are performing competitively, and yet significant areas of distress still exist in those places (Berube & Murray, 2018). This illustrates that the relationships among economic change, demographic shifts, public policy, and urban redevelopment are complex.

The multi-scalar nature of the macrostructures and microprocesses associated with urban development, therefore, require careful attention to spatial patterns of population, the economy, and governance at the metropolitan, county, city, and other sub-state and municipal levels. Indeed, for over a century, scholars have labored to understand the complex relationships among the global economic order, national policies, urban politics, civic cultures, and community action and the corresponding uneven pattern of urban growth, decline, and quality of life across the scales of the nation, regions, metropolitan areas, cities, neighborhoods, and even city blocks—the geographical context of urban development (Huggins & Thompson, 2017). This requires a robust analytical framework and broad base of empirical data that supports sophisticated spatial analysis.

### 1.2. Important Geographic and Population Characteristics Linked to Economic Development

Many important spatial characteristics of metropolitan regions—such as land use and population settlement patterns—are linked to one another and to economic outcomes. After a review of the literature, and considering our overarching research objectives, we consider five characteristics that vary inter-regionally and are thought to impact upon economic growth and vulnerability, albeit in ways that show mixed correlations: fragmentation, sprawl, racial segregation, economic inequality, and poverty. Many variables are also nested in a contrast between city or county boundaries and census regions, owing to the use of such measures in past work (Beauregard, 2012; Hill, St. Clair et al., 2012).

Fragmentation dilutes and separates out the resources available to a region. Decades ago, Hill (1974) was criticizing political fragmentation for many of America’s urban challenges. Others have subsequently remarked on how the antitheses of consolidation or collaboration are important for promoting economic competitiveness (Cooke & Morgan, 1998; Martin, Kitson, & Tyler, 2012; Scott, 1998; Storper, 1997). Fragmentation has been positively correlated to all of our other characteristics. More fragmented governance leads to greater sprawl, as entities seek to compete against one another by authorizing surplus land for development (Razin & Rosentraub, 2000). Fragmentation also contributes to differentiation in racial composition of municipalities (what we operationalize here as segregation) and socioeconomic status, making inequalities explicit in the socio-spatial landscape (Rusk, 2003; Weiher, 1991). Fragmented regions “perpetuate if not intensify the racial, ethnic, and class differences that have long been the bane of the large US metropolis” (Morgan & Mareschal, 1999, p. 579).

Sprawl is less clearly connected to our other characteristics under consideration. More sprawl can exacerbate inequality via the socio-spatial differentiation of populations into far-flung suburbs and isolated inner-cities (Jargowsky, 2002; Rusk, 1993; Wheeler, 2006). And while, increasingly, levels of poverty in the city are connected with middle-class residents moving outwards, sprawl can multiply regional poverty and strain public services as municipalities struggle to maintain the legacy costs of excess infrastructure or, alternatively, the demands of new residents (Orfield, 1997, 2002; Wiewel & Schaffer, 2001). But sprawl’s connections to racial segregation are less clear. Denser urban regions beget sprawl via a desire to demarcate clear racial boundaries; in contrast, lower density regions may yield less direct perceived effects from integration (Galster & Cutsinger, 2007). Sprawl can of course also be used as a tool to escape racial integration, especially by way of covertly racist housing practices (Farrell, 2002) and in inelastic regions, where annexation is no longer a viable option (Rusk, 1993).

Racial segregation has clear links to economic inequality and poverty. On both accounts, the notion is that spatial differentiation of populations by race occurs in regions that are more unequal and poorer (Ananat, 2011; Bresson, Madre, & Pirotte, 2004; Massey & Fischer, 2000). This is thought to be both cause and effect: Separating population by race may reflect existing inequalities and regional economic status, but this separation also makes new investments more tenuous. Likewise, economic inequality and poverty are often linked to one another within the US (Friedman & Lichter, 1998): While a region can be more or less equitable, the fortunes of a region and its relative distribution of wealth are associated.

Thus, overall, a general argument can be made that all five of these characteristics of urban spaces are positively correlated to one another. The assumption that these all impact upon one another the same from one region to another is a simplification, however, and the desire to make this distinction drives our motivation for this article. In addition, however, each of these impacts upon economic vulnerability in different ways that must also be considered.

The relationship between fragmentation and economic growth is mixed. While some provide support for Tiebout’s hypothesis that more fragmented regions foster competition and increase prosperity (Akai & Sakata, 2002; Grassmueck & Shields, 2010), others are more apprehensive (Hendrick, Jimenez, & Lal, 2011; Stansel, 2005). Long-term fiscal liabilities that result from fragmented governance may have a negative feedback loop on local development and economic opportunity (Highsmith, 2015; Sadler & Highsmith, 2017). Similarly, although sprawl itself is an outcome of economic growth, its proliferation is often found to be at odds with prosperity (Burchell, 1997; Ciscel, 2001). In other words, regions that grow with less sprawl are considered to be more competitive for continued future growth.

Racial segregation, inequality, and poverty are all negatively correlated to economic growth (Hobor, 2013; Li, Campbell, & Fernandez, 2013; Pastor & Benner, 2008; Sunley, 2000). The links to inequality and poverty are perhaps more intuitive, given their direct connection to the presence of well-paying jobs. But the link to racial segregation is, as noted above, possibly an outgrowth of how institutions are organized and policies are implemented in ways that have racist implications (Tighe & Ganning, 2015), all of which ultimately make regions less competitive.

The further spatial consideration then is around the relationship between the central city and the wider region. Prior studies include comparisons of a variety of measures between city or county boundaries and census regions (Beauregard, 2012; Hill, St. Clair et al., 2012) as a means of accounting for the uneven patterns of regional economic development and capturing the differences between core cities and their surrounding geographic areas, usually analyzed as their suburbs, periphery, or outer cities (Markusen & DiGiovanna, 1999; Orfield, 2002). In fact, more than having different characteristics, the trajectories of growth and decline in central cities and metropolitan areas are linked (Squires & Kubrin, 2005). In regards to the actual relationships across cities and regions, however, they are not clearly understood and deserve greater scrutiny (Farole, Rodríguez-Pose, & Storper, 2011; Huggins, 2016).

### 1.3. Study Context

This study incorporates data from metropolitan areas, counties, and central cities utilized in recent scholarship. We initially combined the conventional set of distressed, weak-market or shrinking US cities and urban regions defined by economic and population measures (Beauregard, 2009; Hartt, 2018; Pastor & Benner, 2008; Wolman et al., 2008) with the group of places that have a common legacy of extensive deindustrialization (Berube & Murray, 2018; Booth, 1986). Our initial limiting factor was to include only Metropolitan Statistical Areas from four census regions (representing seven divisions) to capture wider geographic factors associated with economic vulnerability (Hill, Wolman et al., 2012). Prior research found that the West region had significantly less economic stress in metropolitan areas (St. Clair, Wial, & Wolman, 2012). Further, of the 79 counties that experienced a substantial shift of more than 20% in manufacturing share of employment from 1970 to 2016 (Berube & Murray, 2018), 76 are outside the West region. The goal of the parameters is to be able to compare more and less successful places among and within common economic, cultural, and political contexts.

Given the ways that such phenomena overlap in economic regions, we present here the creation of a Prosperity Risk Index for evaluating Multi-scalar Economic Development and equity patterns (PRIMED) and investigate its relationship to regional economic vulnerability. Given that census regions have been used as a dummy variable in past work, we are similarly framing effects from that level (Hill, St. Clair et al., 2012). Our sample includes mid-sized urban regions in the Eastern US—here defined as areas in the Northeast, Midwest, and South census regions—and having populations between 250,000 and 2,500,000 (N = 131). Fourteen regions were removed because of missing Brookings data, leaving 117 in the final sample. Initial investigation revealed heterogeneity in the data for the South census region, and it had twice as many cities as the other regions (65 compared to 20 in the Northeast and 32 in the Midwest). For our subgroup analysis, therefore, we subdivided the South into the South Atlantic census division (n = 30) and a South Central grouping that included the East South Central and West South Central census divisions (n = 35).

Our key research questions are two-fold: 1) Do land use and population-based characteristics making up the PRIMED predict economic prosperity or vulnerability? 2) How do these relationships vary across regions? Our hypotheses are that PRIMED characteristics will broadly correlate with measures of economic vulnerability, and that regional differences attributable to land use policy and economic trajectory will lead to differences in how the PRIMED manifests itself on economic vulnerability.

## 2. Methods

Our article revolves around the central concept that various land use and population characteristics impact upon regional economic vulnerability. As such, we leverage data from the US census (population and socioeconomic characteristics), census boundary shapefiles, and county-level data from the Brookings Institute on economic development that point to our construct of economic vulnerability.

US census data were downloaded at the census tract level for every mid-sized urban region in the Eastern US. Key variables included population by race, as well as four variables commonly used as proxies for material and social deprivation, which included the percent of: people living in poverty, adults with low educational attainment, people in the workforce who are unemployed, and lone parent families. A composite socioeconomic distress index was computed by taking the sum of the z-scores of these four variables (as in Pampalon, Hamel, Gamache, & Raymond, 2009). Average distress scores were then appended to each level of geography (noted immediately below) for which analyses were conducted. Glaeser, Scheinkman, and Shleifer (1995) used similar measures—including income, unemployment, and educational attainment—in a study of economic growth.

**Figure 1.**
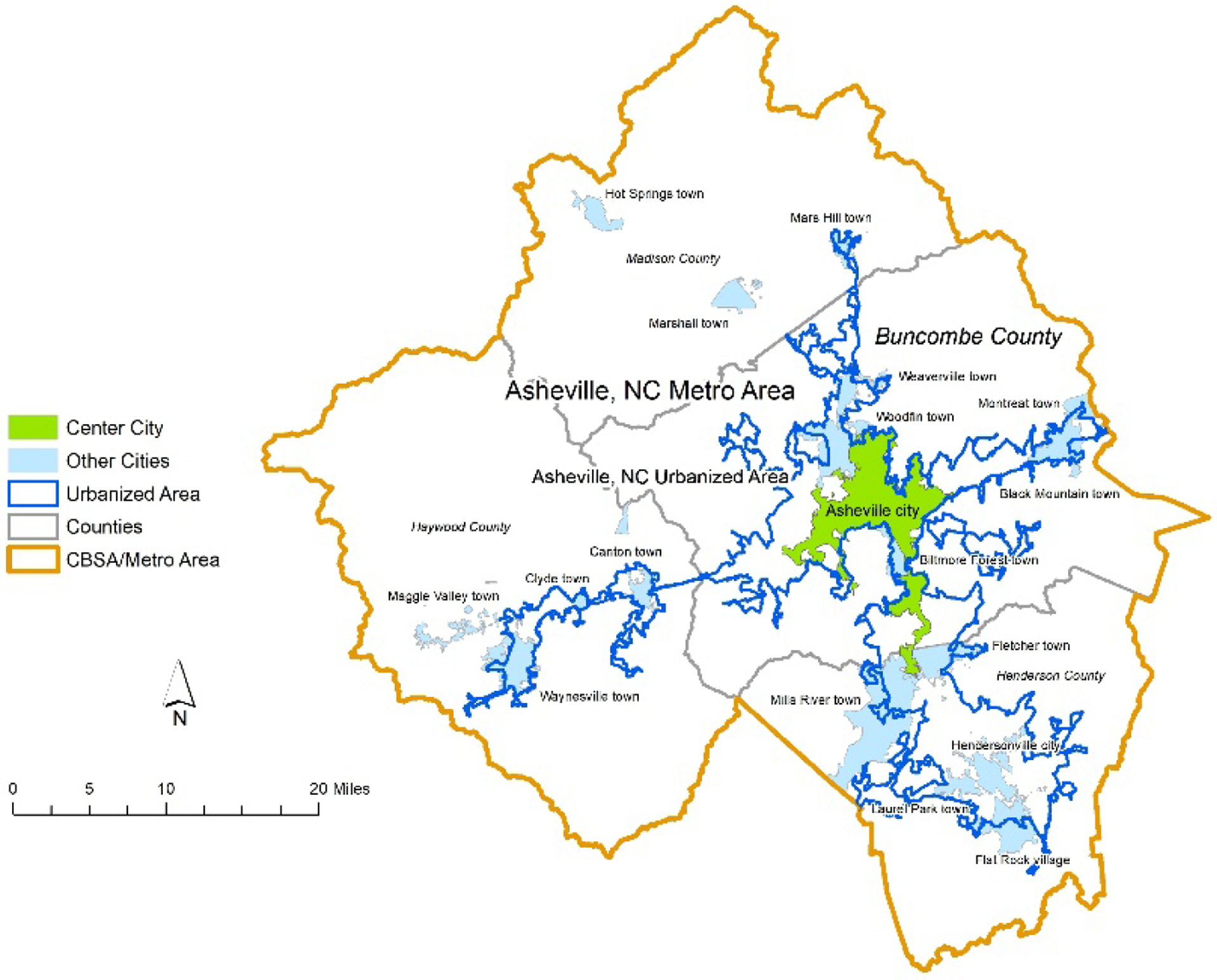
Example of city-urban area-county-CBSA.

In addition to compiling sociodemographic data for census tracts, census boundary shapefiles included cities, counties, urbanized areas, and core-based statistical areas (CBSAs). We differentiated between center cities (the largest city in the CBSA) and other incorporated municipalities outside of the center city. For each center city, we linked the county of which it was a part, or which immediately surrounded it (e.g., in the case of independent cities in Virginia). The urbanized area of each center city is a census-defined metric indicating the densely settled land area in a region, regardless of whether or not it falls inside of an incorporated municipality. The CBSA represents one or more counties that encompass a metro region’s commuting zone. An example of the differentiation is shown in Figure 1.

### 2.1. Construction of Indices

With data appended at multiple levels, we then treated each city-county-urbanized area-CBSA set as a one-to-one join. That is, only the largest city in a metro area is included in our analysis, and is linked to its corresponding county, urbanized area, and CBSA. As a result, mid-sized cities within the metro area of big cities (e.g., Kenosha outside of Milwaukee, or Troy outside of Detroit) were not included. For each of the five characteristics noted above (fragmentation, sprawl, segregation, inequality, and poverty) and the measure of economic vulnerability, we created an index. Each index is a composite of the sum of z-scores for constituent variables. Variables were chosen based on their past use, and on the potential utility of explaining the phenomenon in question.

#### 2.1.1. Fragmentation

The fragmentation index is comprised of three variables: the number of municipalities per 10,000 population (Dolan, 1990; Hendrick et al., 2011; Ostrom, Parks, & Whitaker, 1974; Stansel, 2005), the percent of the center city population in the CBSA, and the percent of the center city population in the urbanized area (Foster, 1993; Stansel, 2005). Ours reflects earlier indices like the one created by Grassmueck and Shields (2010) but makes use instead of municipalities’ land use decision-making power rather than political and economic power.

#### 2.1.2. Sprawl

The sprawl index has five variables: the ratios of three population densities (urbanized area vs. center city, CBSA vs. urbanized area, and outside of cities vs. inside cities) and two land areas (center city vs. urbanized area and all cities vs. urbanized area). Frenkel and Ashkenazi (2008) recommended measures of density and compactness, while Ruiz, Cuevas, Braçe, and Garrido-Cumbrera (2018) offered that an ideal measure of sprawl would take a sub-municipal measure into account. Kew and Lee (2013) used National Land Cover Datasets to measure changes in the amount of developed land. Our use of only census data in calculating population densities and land area ratios is to highlight the utility of that singular dataset. On the land area side of the equation, Morgan and Mareschal (1999) used a measure of land area in the center city.

#### 2.1.3. Racial Segregation

The racial segregation index signifies the difference in the percentages of white and non-white residents at three levels: CBSA vs. center city, urbanized area vs. center city, and county vs. center city. Although this contrasts with the use of segregation indices constructed for *within* cities, our purpose here is to construct indices with similarities to one another, and across municipalities in a metro region.

#### 2.1.4. Economic Inequality

The economic inequality index is similar in composition to the segregation index, but instead of race considers differences in socioeconomic distress at three levels: CBSA vs. center city, urbanized area vs. center city, and county vs. center city. Logan and Schneider (1982) used a ratio of suburban to center city income in calculating regional inequality; this forms a primary motivation behind our index.

#### 2.1.5. Poverty

The poverty index is similarly composed, but instead of the difference between center city and other geographic units, it is simply the composite of center city, urbanized area, and county socioeconomic distress.

#### 2.1.6. Economic Vulnerability

Every city and metropolitan area is a dynamic economic center that changes over time. To assess a general level of economic vulnerability, data is drawn from a Brookings report that examined recent changes in population and employment at the county level within a context of changing economic sector mix and performance against national trends (Berube & Murray, 2018). Population change alone is a strong indicator of both growth and decline, as the analyses of shrinking cities has determined (Hartt, 2018; Pallagst, Wiechmann, & Martinez-Fernandez, 2013; Richardson & Nam, 2014; Weaver, 2017). The change in share of manufacturing employment from 1970 to 2016 points toward the strength of the longer waves of economic and social change that have been part and parcel of deindustrialization. Cities, counties, and metropolitan areas that have experienced a high level of deindustrialization have developed uniquely challenging circumstances with urban form (Ryan, 2012), culture (Dewar & Thomas, 2013), and human capital (van Agtmael & Bakker, 2016).

### 2.2. Analysis

Our analytical plan includes a multi-level modeling approach, nesting cities within their constituent census groupings (Northeast, Midwest, South Atlantic, South Central) and controlling for sociodemographic characteristics, to determine the independent influence of our land use and population-based indices on economic vulnerability.

## 3. Results

### 3.1. PRIMED Characteristics

The PRIMED was composed of the unweighted average of the z-scores for each of the five land uses and population characteristics. For each of the four census groupings, the average z-score for the five characteristics and the PRIMED are shown in Table 1 below. The Northeast is the most fragmented, sprawling, segregated, and unequal grouping, while the South Central is the most poverty-stricken. Conversely, the South Central is also the least fragmented and sprawling. The South Central and South Atlantic are tied for the least segregated, while the Midwest is the least unequal. Overall, the Northeast has the highest PRIMED score (denoting prosperity risk), while the South Central has the lowest.

**Figure 2.**
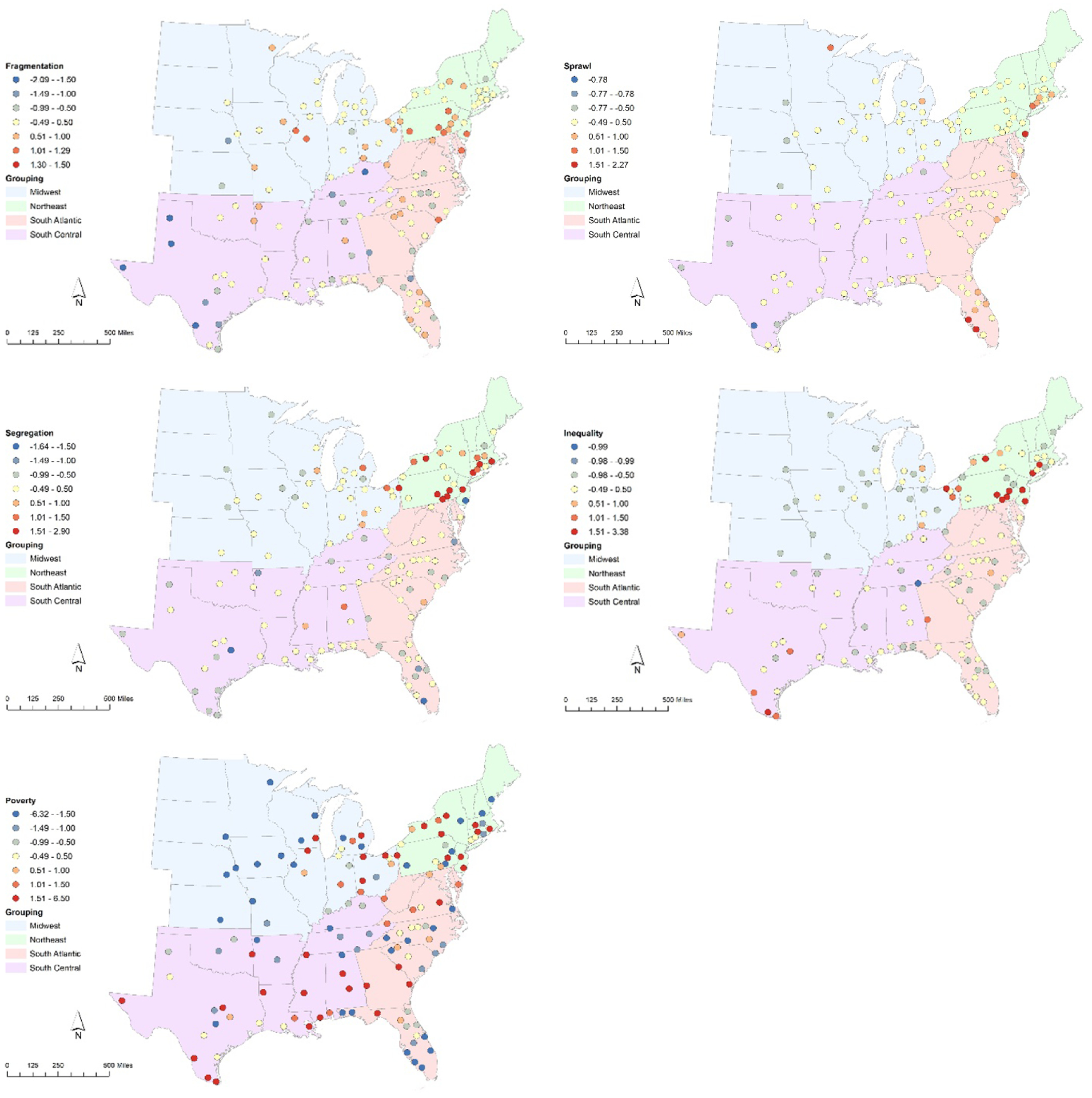
PRIMED characteristic z-scores for each urban region.

The PRIMED characteristics scores for each region in our sample are also illustrated in Figure 2, with counties and states colored by their respective census divisions and regions colored by the PRIMED score (with blue shades representing lower risk scores and red shades representing higher risk scores). Generally, higher risk scores are seen in the Northeast and Eastern Midwest, while lower risk scores can be found throughout the study area.

The most fragmented urban regions tend to fall in the Northeast and South Atlantic, while the least fragmented areas are typically in the South. The sprawl index did not have as many large deviations from the mean, with most areas falling within half a standard deviation (yellow in the map). Regions with more sprawl out from the center city can be found in Florida, up the Atlantic coast, and in a couple spots in the Midwest. Regions with less out-of-city sprawl, by contrast, tend to be out west. As with the fragmentation index, the most segregated regions are in the Northeast, while less fragmented urban regions can be found throughout. Center-to-out-of-center city economic inequality is highest in the Northeast and along the Rio Grande Valley in Texas. Lower inequality can be found throughout the Midwest and South. Overall regional poverty saw the biggest deviations from the mean, with most falling at least one standard deviation away. Unlike the clear spatial/regional patterns for the other indices, no clear pattern is seen with poverty: wealthy regions about poorer regions throughout the study area.

Our motivation is not only to illustrate regional differences, but to highlight exemplars within regions as well. As another descriptive result, Tables 2 and 3 show the least and most municipally fragmented, geographically sprawling, racially segregated, economically unequal, and poverty-stricken regions in each census grouping (with their ranking shown in parentheses). The general pattern supports the base hypothesis: that cities suffering more from economic decline appear more frequently in the ‘most’ categories (Table 2), while cities that have more recently been growing appear in the ‘least’ categories (Table 3).

**Figure 3.**
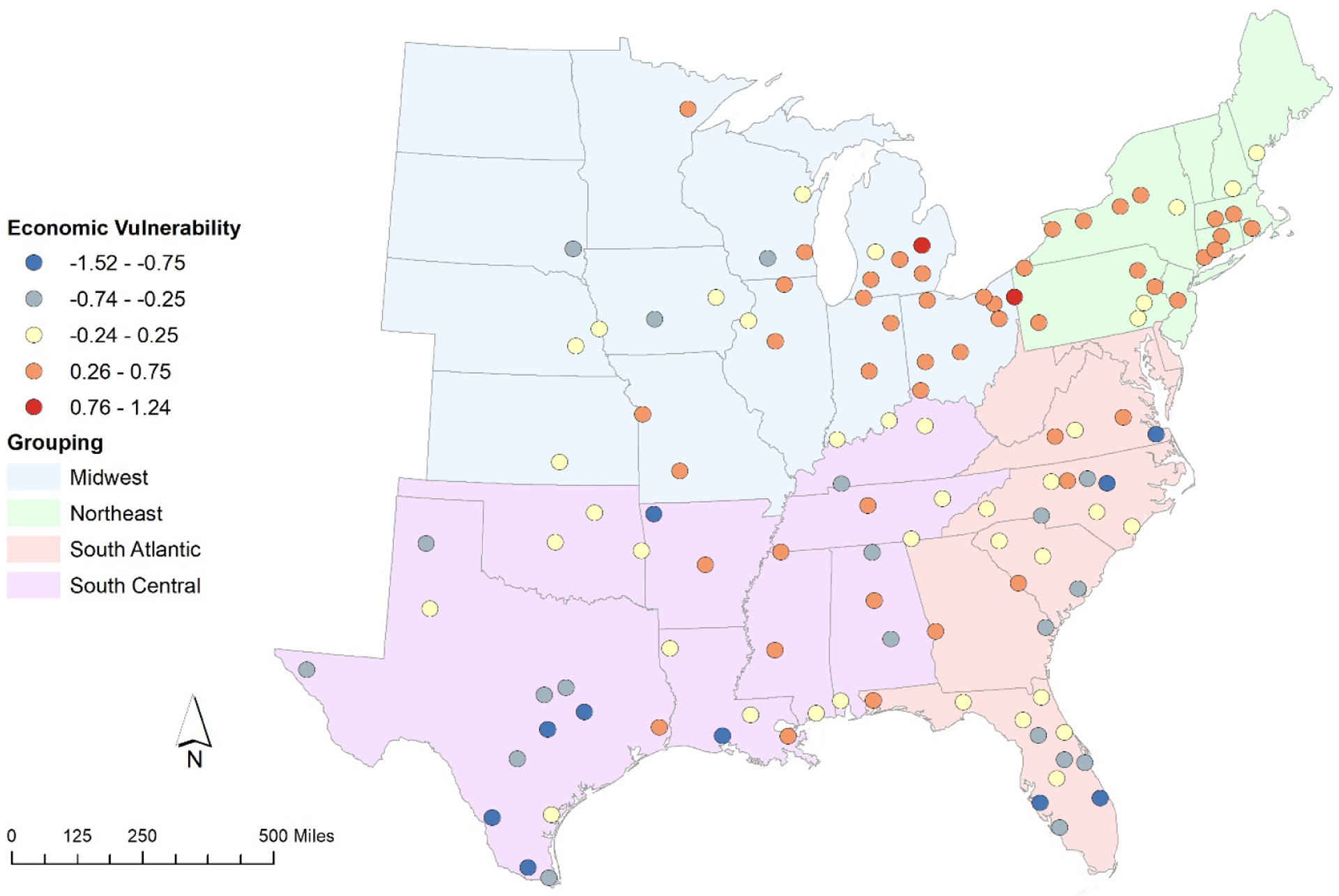
Economic vulnerability z-scores for each urban region.

As with the PRIMED characteristics in Figure 2 above, the economic vulnerability index is illustrated spatially in Figure 3. Blue shades once again signify lower vulnerability, while red shades represent higher vulnerability. The pattern of high scoring areas for vulnerability is similar to that for the prosperity risk indices, being primarily concentrated in the Northeast and Midwest. In contrast, however, vulnerability is more consistently low in the South Atlantic and South Central groupings. By our measure, all of the lowest vulnerability regions lie in the south. Regionally, the most economically vulnerable urban regions are: Scranton, PA, in the Northeast (9 overall); Flint, MI, in the Midwest (1); Columbus, GA, in the South Atlantic (10); and New Orleans, LA, in the South Central (4). Conversely, the least economically vulnerable urban regions are: Manchester, NH (55 overall); Sioux Falls, SD (19); Virginia Beach, VA (5); and McAllen, TX (1). In our discussion, we introduce key outliers in each region that may yield meaningful inquiry in future work.

### 3.2. Regression Models

In pursuit of our main objective to illustrate regional differences in the relationship between our prosperity risk indices and the economic development outcomes (low levels of which point to economic vulnerability), we illustrate here the results of a series of linear regression models. The first is a regression model without interactions that omits region as a covariate (to reflect overall trends). The second is an interaction model that includes region and an interaction term between each characteristic in the PRIMED and region to capture differences between regions in these trends. To ensure the indices in the PRIMED reflected different constructs and were acceptable for both of these models, we ran a series of diagnostic tests. A check for multicollinearity revealed acceptable VIFs (all below 2.5), while the residuals followed an approximately normal distribution with reasonable homoscedasticity.

Table 4 shows the results of the first regression model linking the PRIMED indices to economic vulnerability. The model illustrates significant positive associations with segregation (β = 0.42, p <0.001) and poverty (β = 0.05, p = 0.01; e.g., more segregation and poverty equate with more economic vulnerability), and a significant negative association with inequality (e.g., more inequality equates with less economic vulnerability, echoing Rusk, 1993). While not significant, fragmentation is slightly positively associated and sprawl is slightly negatively associated with economic vulnerability.

Although these overall relationships are instructive for viewing the connection between the PRIMED indices and economic vulnerability, it is harder to see exactly what is happening—and the direction of the associations—within each region. In this step, an ANOVA was run (with results shown in Table 5) to determine if there were significant differences between regions for each PRIMED index. We can observe that significant differences exist between regions (F = 14.089, p < 0.001), and (in agreement with the results in Table 4) in rates of segregation (F = 15.058, p < 0.001) and poverty (F = 10.175, p < 0.01). The interaction model suggests that segregation is strongly significantly different between regions (F = 5.323, p < 0.01), while sprawl is marginally significant between regions (F = 2.584, p = 0.058).

**Figure 4.**
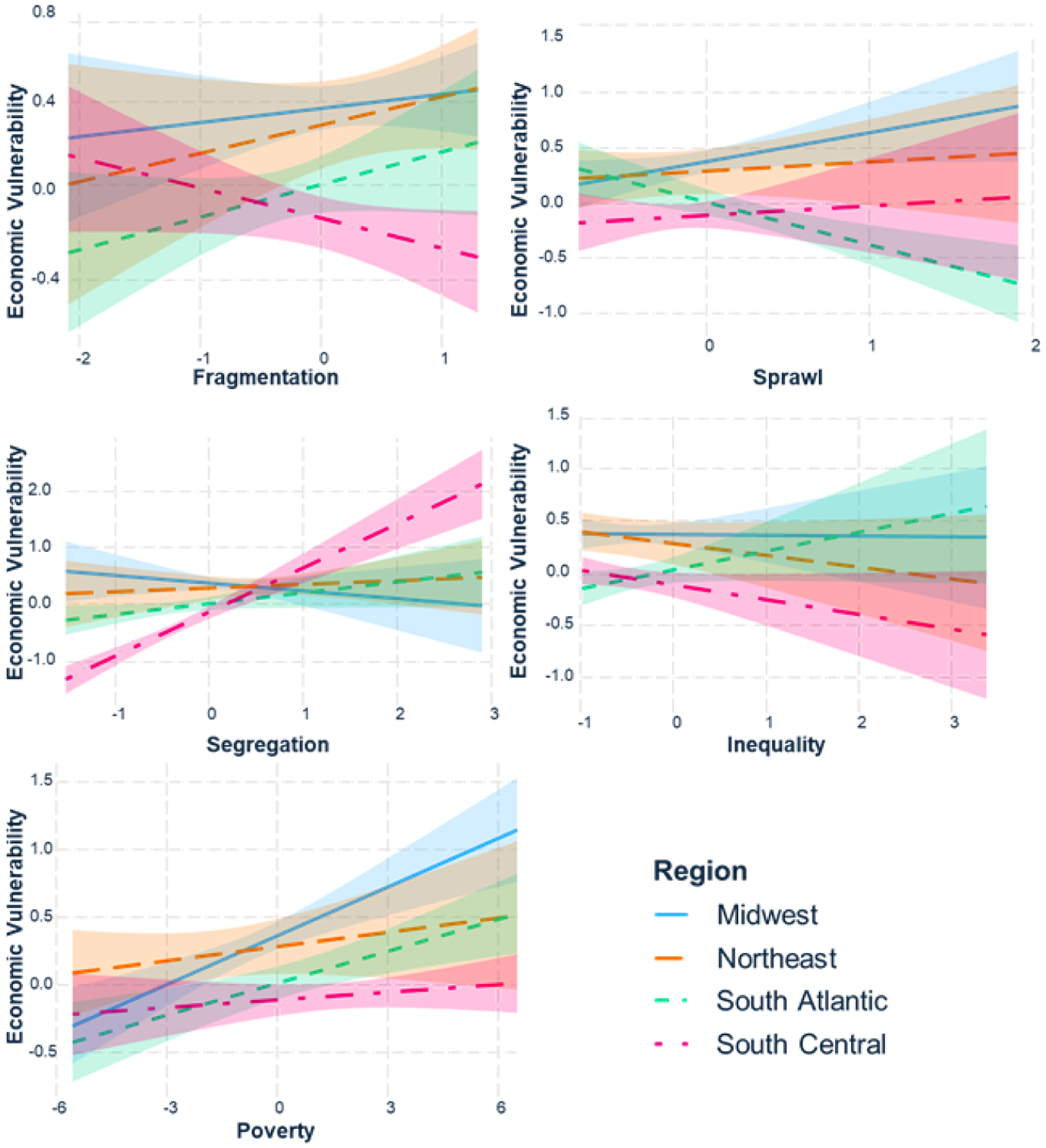
Subgroup plots by region for interaction model.

Figure 4 highlights the slopes for each region between the 5 PRIMED indices and economic vulnerability. Fragmentation is positively associated with economic vulnerability in the Midwest, Northeast, and South Atlantic. But in the South Central, less fragmented regions connote more economic vulnerability. The general pattern is similar for sprawl. In the Midwest, sprawl is significantly positively associated with economic vulnerability. It is less strongly but slightly positively correlated in the Northeast and South Central, but in the South Atlantic, more sprawl connotes less economic vulnerability.

Racial segregation is generally not a strong predictor of economic vulnerability in the Midwest, Northeast, and South Atlantic, but is strongly correlated to economic vulnerability in the South Central. Economic inequality is only positively correlated to economic vulnerability in the South Atlantic. Elsewhere (particularly in the South Central), more economic inequality connotes less economic vulnerability. Poverty is very significantly positively correlated with economic vulnerability in the Midwest, but only marginally significant in the other regions.

From examining the subgroups in Table 6, we see the main differences between regions for segregation occur in the South Central region, where segregation is strongly positively associated with economic vulnerability (β = 0.78, p < 0.01), while the other regions are weakly associated. Further, the differences with respect to sprawl are due to the South Atlantic being strongly negatively associated with economic vulnerability, while the other regions have a positive association between sprawl and economic vulnerability.

By introducing these interaction terms, some of the variance originally explained by inequality is being explained in the interaction terms, and inequality is no longer a significant predictor. We note that the significant differences between regions in segregation are accounted for by the South Central’s strong positive association to economic vulnerability, while the association is weak or slightly negative in the South Atlantic, Midwest, and Northeast.

## 4. Discussion

### 4.1. National Trajectories and Regional Variations in Prosperity Risk

Our article presents data and analysis that describes important geographic characteristics influencing economic performance and vulnerability, namely: fragmentation, sprawl, segregation, inequality, and poverty. We used census data to compute indices for each of these characteristics and combined them into an index we call the PRIMED. We then examined the relationship among the PRIMED sub-indices and economic vulnerability and found the model illustrates significant positive associations with segregation and poverty (e.g., more segregation and poverty equate with more economic vulnerability), and a significant negative association with inequality. This affirms the general trajectories of urban areas in which high economic growth is linked with low inequality, termed positive development, and vice versa (Rusk, 1993).

At the same time, the factors contributing to positive development vary across regions. We observe that significant differences exist between regions (F = 14.089, p < 0.001), and (in agreement with the results in Table 4) in rates of segregation (F = 15.058, p < 0.0001) and poverty (F = 10.175, p < 0.01). The interaction model suggests that segregation is strongly significantly different between regions (F = 5.323, p < 0.01) marginally while sprawl is significant between regions (F = 2.584, p = 0.058).

These findings illustrate that the barriers to positive local economic development manifest in dissimilar forms in each region. In the Midwest, Northeast, and South Atlantic fragmentation and sprawl, to varying degrees, are positively associated with economic vulnerability. But in the South Central area, less fragmented regions and less sprawl connote more economic vulnerability. Furthermore, the main differences between regions for segregation occur in the South Central region, where segregation is strongly positively associated with economic vulnerability (β = 0.78, p < 0.01), while the other regions are weakly associated. These findings are valuable for local economic development practitioners who may be seeking further contextual information on their region, or for others interested in understanding the dynamics that drive regional competitiveness and prosperity.

### 4.2. Metropolitan Dynamics and the Place of the Central City

Generally, the prosperity risk index trended in the same direction as our index of economic vulnerability, contextualizing regional differences in the influence of specific characteristics on vulnerability. Yet not all urban areas neatly fit into the regression line. For example, in Figure 4, the South Central region is distinct from the others in terms of fragmentation and segregation, with fragmentation being negatively correlated and segregation being positively correlated to economic vulnerability. Factors such as date of urban development, differences in rights and responsibilities of municipalities, and regional variation in economic trajectory may all be potential explanations for this regional outlier effect.

Here we briefly explore two pairs of outliers where regions were ranked ‘high’ in one index but not the other. Examining a pair of outliers by region illustrates the relationships among variables for particular places but also points to the limitations of the quantitative analysis and brings into focus the policy and institutional features shaping these characteristics that may be driving the differential between the prosperity risk and economic rank at the individual metro scale of analysis.

#### 4.2.1. Example 1: Northeast

In general, the Northeast is the most fragmented, sprawling, segregated, and unequal, and therefore, the region has the highest PRIMED score (0.53). In the regional interaction model highlighted in Figure 4, we found fragmentation and sprawl were positively associated with economic vulnerability in the Northeast. The Bridgeport, CT, and Allentown, PA, urban areas are both outliers to the general national pattern of how prosperity risk and economic vulnerability are correlated. Bridgeport is over-performing economically (74/117) given its very poor prosperity risk (109/117; Table 2).

One distinctive feature of Bridgeport is that it is contained within one county, which may create a public environment that cuts against the trend of fragmentation being positively associated with economic vulnerability. The city is not viewed as having reinvented itself from its industrial peak, but with significantly less governmental complexity, it may have enabled economic development activities to be conducted within a single policy framework with feedback loops among economic activity, tax revenue generation, service provision, and quality of life. For instance, the leading economic development voice for the region, the Bridgeport Regional Business Council, explicitly recognizes the need for increasing the county’s grand list (Connecticut’s term for tax base), whereas most business-led economic development agencies prioritize reducing tax burdens (Bridgeport Regional Business Council, n.d.).

Allentown has higher economic vulnerability (76/117), despite a low prosperity risk ranking (36/117). Allentown has relatively lower levels of poverty and inequality, but slow population growth as a legacy city is likely dragging down economic revitalization. Indeed, the area has experienced extreme deindustrialization since 1970: the percent of jobs in manufacturing in Lehigh County declined from 58% to 12% in 2016 (Berube & Murray, 2018). Compared to the single-county geography of the Bridgeport area, the four counties that now constitute the urban area also extend into New Jersey; this bi-state orientation deserves greater scrutiny.

#### 4.2.2. Example 2: South Central

The South Central group has the lowest average PRIMED z-score (−0.56). Although the South Central has the most impoverished urban areas, it is also the least fragmented and least sprawling by our metrics. The group likewise has relatively less segregation and economic vulnerability. Our regional analysis identified that economically vulnerable regions are less fragmented but more racially segregated. Waco, TX, and Little Rock, AR, are interesting outliers in the South Central. While Waco has noticeably less economic vulnerability (75/117) than prosperity risk (16/117), Little Rock is the reverse (53/117 in economic vulnerability, 89/117 in prosperity risk).

Waco contrasts with the regional pattern where less fragmentation and more racial segregation lead to economic vulnerability. One factor that may be supporting better economic outcomes in the Waco area that is not evident in the indices is the recent growth of Baylor University. Universities, especially research-intensive universities and other anchor assets in central cities, have positive effects on regional employment as well as local housing markets (Ehlenz, 2019). Indeed, Bagchi-Sen and Smith (2012) conclude that universities play a significant role in their regions. The post-recession revitalization efforts in Waco that have included Baylor University would not likely be reflected fully in our analysis.

Segregation is higher in Little Rock than in Waco, and there is more economic vulnerability despite an overall higher PRIMED score. The Little Rock area has been successful to a point in diversifying from an industrial and manufacturing economy, but transitioning to a knowledge-based economy has been challenging in Arkansas overall (National Research Council, 2012). The design of the University of Arkansas system is such that fewer than 25% of enrolled students are in the Little Rock area, with the flagship University of Arkansas in Fayetteville accounting for nearly half of all students and a majority of research activity (University of Arkansas System, n.d.). Certainly, other private and public institutional factors are shaping the fortunes of individual metropolitan regions and this deserves attention in future research.

### 4.3. Implications for Economic Development Practice

Economic development practice should acknowledge that spatial patterns of economic growth and social equity are linked at the national, regional, and metropolitan scales. This affirms the value of the panoply of development approaches that seek to simultaneously achieve better outcomes economically and socially for a broad base of people and places within an urban area. Furthermore, the results of the interaction model highlighting how PRIMED characteristics vary inter-regionally may have practical benefits for those interested in understanding the dynamics driving competitiveness and prosperity in a particular place. For instance, in the South Central region as noted above, the higher rate of racial segregation is associated with more economic vulnerability. Therefore, as Li et al. (2013) have argued, in these cases a need may exist for mobility policies that support non-White and lower income households as part of comprehensive economic growth strategies. Future work should therefore make use of this and related indices to study a more fine-grained approach whereby researchers develop deeper understandings of what leads some regions to succeed while others do not.

One disconcerting result of our analysis of 117 mid-sized urban regions is the spatial clustering of areas in the bottom third of both indices. These include 29 regions from 20 different states, including both a well-known group of places in the Midwest and Northeast such as Buffalo, NY, Flint, MI, Trenton, NJ, and Youngstown, OH, that have experienced high levels of deindustrialization, and a number of others in the South such as Birmingham, AL, Jackson, MS, Memphis, TN, and Richmond, VA. Part of our larger research agenda is to analyze additional quantitative and qualitative data layers relative to regional inequality and economic vulnerability at closer scales. For instance, the PRIMED results raise questions about relevant state, regional, county, and city public policy and fiscal features in the most vulnerable and fragmented metropolitan areas and how institutions and networks performing economic and community development map onto the political geography (Rusk, 1993). Further assessments will illuminate the distinguishing relationships between metropolitan sociodemographic characteristics, spatial patterns, and development dynamics. Indeed, the presence of these diverse urban areas at the bottom of both indices suggests a direction for future inquiry and serves as a prompt for further research.

Interestingly, places with varied economic histories including Augusta, GA, Birmingham, AL, Memphis, TN, and Richmond, VA, in the South were also in the bottom third of both indices. One implication for economic development in older industrial cities is that the barriers to growth in the 21st century may be less about historical economic patterns and more about the socio-spatial relationships that exist today. Likewise, a broader set of urban areas is struggling with the challenges often associated with deindustrialization, and policy and institutional innovation around equitable development is needed in socially divided and economically depressed areas.

## 5. Conclusion

The development and equity patterns of metropolitan urban areas are of great consequence for people and for local communities. Seeing that it is not only feasible, but prevalent, for places to achieve both high levels of economic performance and low levels of prosperity risk opens up numerous paths for policymakers, planners, and practitioners in local economic development to pursue. The fact that relationships exist among the 5 PRIMED characteristics and economic vulnerability has implications for public health, not least because of the links between inequality and poverty on the one hand and health outcomes on the other. Moreover, the particular regional and metropolitan patterns may guide interventions to be more effective than reliance on national trends alone.

Our inter- and sub-regional analysis of urban areas highlights the multi-scalar geographic context in which uneven development processes literally take place. At the same time, the further examination of individual urban areas illustrates the importance of taking stock of public and private institutions at the metropolitan, county, city, and local scales. In reality, the construction of social, political and cultural scales at the metropolitan, local, and neighborhood levels are also important elements to consider, especially regarding how they shape economic relations and spatial contexts (MacKinnon, Cumbers, Pike, Birch, & McMaster, 2009). The central challenge for urban geographers is “understanding the role and significance of the shifting array of actors and institutions shaping urban social, political, and economic geographies” (Elwood, 2005, p. 262). Ultimately, we believe the PRIMED and these findings are valuable for local economic development practitioners who may be trying to better understand the dynamics of their own or comparable urban regions. Likewise, the PRIMED can be used in future research aimed at understanding the dynamics behind urban planning that may drive regional competitiveness and prosperity.

## Figures and Tables

**Table 1.** Z-scores for PRIMED characteristics by census grouping.

	Fragmentation	Sprawl	Segregation	Inequality	Poverty	Prosperity Risk
Northeast	0.46	0.17	0.82	0.80	0.31	0.53
Midwest	0.13	−0.08	0.04	−0.30	−0.53	−0.14
South Atlantic	0.02	0.16	−0.28	−0.27	−0.59	−0.19
South Central	−0.45	−0.22	−0.28	−0.10	0.87	−0.56

**Table 2.** Highest scoring urban regions by census grouping (overall rank in parentheses).

	Fragmentation	Sprawl	Segregation	Inequality	Poverty	PRIMED Overall
Northeast	Scranton, PA (1)	Atlantic City, NJ (1)	Hartford, CT(1)	Hartford, CT(1)	Reading, PA (6)	Reading, PA (1)
Midwest	Davenport, IA (4)	Duluth, MN (5)	Youngstown, OH (10)	Cleveland, OH (10)	Flint, MI (2)	Flint, MI (3)
South Atlantic	Salisbury, MD (3)	Cape Coral, FL (2)	Charleston, SC (22)	Columbus, GA (13)	Augusta, GA (10)	Augusta, GA (16)
South Central	Fayetteville, AR (13)	Fayetteville, AR (20)	Birmingham, AL (16)	McAllen, TX (5)	Brownsville, TX(1)	McAllen, TX (5)

**Table 3.** Lowest scoring urban regions by census grouping (overall rank in parentheses).

	Fragmentation	Sprawl	Segregation	Inequality	Poverty	PRIMED Overall
Northeast	Manchester, NH (21)	Binghamton, NY (16)	Atlantic City, NJ (2)	Norwich, CT (23)	Portland, ME (10)	Portland, ME (10)
Midwest	Lincoln, NE (8)	Lincoln, NE (5)	Ann Arbor, MI (7)	Toledo, OH (2)	Cedar Rapids, IA (2)	Lincoln, NE (1)
South Atlantic	Jacksonville, FL (7)	Tallahassee, FL(10)	Naples, FL(1)	Ocala, FL (3)	Naples, FL (1)	Raleigh, NC (5)
South Central	Laredo, TX (1)	Laredo, TX (1)	College Stn., TX (3)	Chattanooga, TN (1)	Austin, TX (12)	Austin, TX(6)

**Table 4.** Regression model highlighting relationships between PRIMED characteristics and economic vulnerability.

	All Cities
N	117
Intercept	−0.03
	(t = −0.64, p = 0.53)
Fragmentation	0.11
	(t = 1.61, p = 0.11)
Sprawl	−0.16
	(t = −1.56, p = 0.12)
Segregation	0.42 ***
	(t = 6.33, p = 0.00)
Inequality	−0.23 ***
	(t = −3.95, p = 0.00)
Poverty	0.05 **
	(t = 2.80, p = 0.01)
R-squared	0.42

Notes:

** p < 0.001;

** p < 0.01;

* p < 0.05.

**Table 5.** Type II ANOVA for interaction model.

	Sum Sq	Df	F	p
Fragmentation	0.012	1	0.116	0.735
Sprawl	0.036	1	0.347	0.557
Segregation	1.554	1	15.058	0.000
Inequality	0.042	1	0.410	0.524
Poverty	1.050	1	10.175	0.002
Region	4.362	3	14.089	0.000
Fragmentation:Region	0.320	3	1.034	0.381
Sprawl:Region	0.800	3	2.584	0.058
Segregation:Region	1.648	3	5.323	0.002
Inequality:Region	0.328	3	1.060	0.370
Poverty:Region	0.462	3	1.493	0.221
Residuals	9.597	93	NA	NA

**Table 6.** Standardized regression coefficients in subgroup analyses relating PRIMED to economic vulnerability.

	Northeast	Midwest	South Atlantic	South Central
Intercept	0.29	0.38	0.02	−0.16
Fragmentation	0.13	0.06	0.15	−0.14
Sprawl	0.08	0.26	−0.39	0.09
Segregation	0.07	−0.14	0.19	0.78
Inequality	−0.11	−0.01	0.18	−0.14
Poverty	0.04	0.12	0.08	0.02
N	20	32	30	35
R-squared	0.58	0.88	0.51	0.51

## References

[R1] Akai N, & Sakata M (2002). Fiscal decentralization contributes to economic growth: Evidence from state-level cross-section data for the United States. : Journal of Urban Economics, 52(1), 93–108.

[R2] Ananat EO (2011). The wrong side(s) of the tracks: The causal effects of racial segregation on urban poverty and inequality. American Economic Journal: Applied Economics, 3(2), 34–66.

[R3] Bagchi-Sen S, & Smith H (2012). The role of the university as an agent of regional economic development. Geography Compass, 6(7), 439–453.

[R4] Beauregard RA (2009). Urban population loss in historical perspective: United States, 1820–2000. Environment and Planning A, 41(3), 514–528.

[R5] Beauregard RA (2012). Growth and depopulation in the United States. In Mallach A (Ed.), Rebuilding America’s legacy cities: New directions for the industrial heartland (pp. 1–24). New York, NY: The American Assembly at Columbia University.

[R6] Benner C (2015). Equity, growth, and community: What the nation can learn from America’s metro areas. Oakland, CA: University of California Press.

[R7] Berube A, & Murray C (2018). Renewing America’s economic promise through older industrial cities. Washington, DC: Metropolitan Policy Program at Brookings.

[R8] Booth DE (1986). Long waves and uneven regional growth. Southern Economic Journal, 53(2), 448–460.

[R9] Bresson G, Madre JL, & Pirotte A (2004). Is urban sprawl stimulated by economic growth? A hierarchical Bayes estimation on the largest metropolitan areas in France. Paper presented at the 10th World Conference on Transport Research, Istanbul, Turkey.

[R10] Bridgeport Regional Business Council. (n.d.). About the Bridgeport Regional Business Council. Bridgeport Regional Business Council. Retrieved from https://www.brbc.org/index.php/home/about-brbc

[R11] Burchell RW (1997). Economic and fiscal costs (and benefits) of sprawl. The Urban Lawyer, 29(2), 159–181.

[R12] Ciscel DH (2001). The economics of urban sprawl: Inefficiency as a core feature of metropolitan growth. Journal of Economic Issues, 35(2), 405–413.

[R13] Cooke P, & Morgan K (1998). The associational economy: Firms, regions, and innovation. Oxford: Oxford University Press.

[R14] Dewar M, & Thomas JM (2013). Introduction: The city after abandonment. In Dewar M & Thomas JM (Eds.), The city after abandonment (pp. 1–17). Philadelphia, PA: University of Pennsylvania Press.

[R15] Dolan DA (1990). Local government fragmentation: Does it drive up the cost of government? Urban Affairs Review, 26(1), 28–45.

[R16] Ehlenz M (2019). Gown, town, and neighborhood change: An examination of urban neighborhoods with university revitalization efforts. Journal of Planning Education and Research, 39(3), 285–299.

[R17] Elwood S (2005). Perspectives on participation, urban research, and the transformation of “local” urban geographies. Urban Geography, 26(3), 261–265.

[R18] Farole T, Rodríguez-Pose A, & Storper M (2011). Cohesion policy in the European Union: Growth, geography, institutions. JCMS: Journal of Common Market Studies, 49(5), 1089–1111.

[R19] Farrell JL (2002). Community development: The FHA’s origins: How its valuation method fostered racial segregation and suburban sprawl. Journal of Affordable Housing & Community Development Law, 11(4), 374–389.

[R20] Foster KA (1993). Exploring the links between political structure and metropolitan growth. Political Geography, 12(6), 523–547.

[R21] Frenkel A, & Ashkenazi M (2008). Measuring urban sprawl: How can we deal with it? Environment and Planning B: Planning and Design, 35(1), 56–79.

[R22] Friedman S, & Lichter DT (1998). Spatial inequality and poverty among American children. Population Research and Policy Review, 17(2), 91–109.

[R23] Galster G, & Cutsinger J (2007). Racial settlement and metropolitan land-use patterns: Does sprawl abet black-white segregation? Urban Geography, 28(6), 516–553.

[R24] Glaeser EL, Scheinkman JA, & Shleifer A (1995). Economic growth in a cross-section of cities (No. w5013). Cambridge, MA: National Bureau of Economic Research.

[R25] Grassmueck G, & Shields M (2010). Does government fragmentation enhance or hinder metropolitan economic growth? Papers in Regional Science, 89(3), 641–657.

[R26] Hartt M (2018). How cities shrink: Complex pathways to population decline. Cities, 75, 38–49.

[R27] Hendrick RM, Jimenez BS, & Lal K (2011). Does local government fragmentation reduce local spending? Urban Affairs Review, 47(4), 467–510.

[R28] Highsmith AR (2015). Demolition means progress: Flint, Michigan, and the fate of the American metropolis. Chicago, IL: University of Chicago Press.

[R29] Hill E, St. Clair T, Wial H, Wolman H, Atkins P, Blumenthal P, … Friedhoff A. (2012). Economic shocks and regional economic resilience. In Weir M, Pindus N, Wial H, & Wolman H (Eds.), Urban and regional policy and its effects: Building resilient regions (pp. 193–274). Washington, DC: Brookings Institution Press.

[R30] Hill EW, Wolman HL, Kowalczyk K, & St. Clair T (2012). Forces affecting city population growth or decline: The effects of inter-regional and intermunicipal competition. In Mallach A (Ed.), Rebuilding America’s legacy cities: New directions for the industrial heartland (pp. 31–79). New York, NY: The American Assembly at Columbia University.

[R31] Hill RC (1974). Separate and unequal: Governmental inequality in the metropolis. American Political Science Review, 68(4), 1557–1568.

[R32] Hobor G (2013). Surviving the era of deindustrialization: The new economic geography of the urban rust belt. Journal of Urban Affairs, 35(4), 417–434.

[R33] Huggins R (2016). Capital, institutions and urban growth systems. Cambridge Journal of Regions, Economy and Society, 9(2), 443–463.

[R34] Huggins R, & Thompson P (Eds.). (2017). Handbook of regions and competitiveness: Contemporary theories and perspectives on economic development. Cheltenham: Edward Elgar Publishing.

[R35] Jargowsky PA (2002). Sprawl, concentration of poverty, and urban inequality. In Squires GD (Ed.), Urban sprawl: Causes, consequences, and policy responses (pp. 39–72). Washington, DC: The Urban Institute Press.

[R36] Kew B, & Lee BD (2013). Measuring sprawl across the urban rural continuum using an amalgamated sprawl index. Sustainability, 5(5), 1806–1828.

[R37] Kodrzycki YK, & Muñoz AP (2015). Economic distress and resurgence in US central cities: Concepts, causes, and policy levers. Economic Development Quarterly, 29(2), 113–134.

[R38] Li H, Campbell H, & Fernandez S (2013). Residential segregation, spatial mismatch and economic growth across US metropolitan areas. Urban Studies, 50(13), 2642–2660.

[R39] Logan JR, & Schneider M (1982). Governmental organization and city/suburb income inequality, 1960–1970. Urban Affairs Quarterly, 17(3), 303–318.

[R40] MacKinnon D, Cumbers A, Pike A, Birch K, & McMaster R (2009). Evolution in economic geography: Institutions, political economy, and adaptation. Economic Geography, 85(2), 129–150.

[R41] Markusen A (2015). Problem-driven research in regional science. International Regional Science Review, 38(1), 3–29.

[R42] Markusen AR, & DiGiovanna S (1999). Comprehending fast-growing regions. In Markusen AR, Lee Y-S, & DiGiovanna S (Eds.), Second tier cities: Rapid growth beyond the metropolis (pp. 3–20). Minneapolis, MN: University of Minnesota.

[R43] Martin R, Kitson M, & Tyler P (2012). Regional competitiveness. London and New York, NY: Routledge.

[R44] Massey DS, & Fischer MJ (2000). How segregation concentrates poverty. Ethnic and Racial Studies, 23(4), 670–691.

[R45] Morgan DR, & Mareschal P (1999). Centralcity/suburban inequality and metropolitan political fragmentation. Urban Affairs Review, 34(4), 578–595.

[R46] National Research Council. (2012). Building the Arkansas innovation economy: Summary of a symposium. Washington, DC: The National Academies Press. 10.17226/13532 23326901

[R47] Orfield M (1997). Metropolitics: A regional agenda for community and stability. Washington, DC, and Cambridge, MA: Brookings Institution Press and Lincoln Institute of Land Policy.

[R48] Orfield M (2002). American metropolitics: Social segregation and sprawl. Washington, DC: Brookings Institution Press.

[R49] Ostrom E, Parks RB, & Whitaker GP (1974). Defining and measuring structural variations in interorganizational arrangements. Publius, 4(4), 87–108.

[R50] Pallagst K, Wiechmann T, & Martinez-Fernandez C (Eds.). (2013). Shrinking cities: International perspectives and policy implications. Abingdon: Routledge.

[R51] Pampalon R, Hamel D, Gamache P, & Raymond G (2009). A deprivation index for health planning in Canada. Chronic Diseases in Canada, 29(4), 178–91. 19804682

[R52] Pastor M, & Benner C (2008). Been down so long: Weak market cities and regional equity. In McGahey RM & Vey JS (Eds.), Retooling for growth: Building a 21st century economy in America’s older industrial areas (pp. 89–118). Washington, DC: Brookings Institution Press.

[R53] Razin E, & Rosentraub M (2000). Are fragmentation and sprawl interlinked? North American evidence. Urban Affairs Review, 35(6), 821–836.

[R54] Richardson HW, & Nam CW (Eds.). (2014). Shrinking cities: A global perspective. Abingdon: Routledge.

[R55] Ruiz DG, Cuevas PD, Braçe O, & Garrido-Cumbrera M (2018). Developing an index to measure sub-municipal level urban sprawl. Social Indicators Research, 140(3), 929–952.

[R56] Rusk D (1993). Cities without suburbs. Washington, DC: Woodrow Wilson Center Press.

[R57] Rusk D (2003). Cities without suburbs: A census 2000 update. Washington, DC: Woodrow Wilson Center Press.

[R58] Ryan BD (2012). Shrinking-city urban form as a determinant of urban policy: The case of Flint, Michigan, USA. Paper presented at the 48th ISOCARP Congress, Perm, Russia.

[R59] Sadler RC, & Highsmith AR (2017). Rethinking Tiebout: The contribution of political fragmentation and racial/economic segregation to the Flint water crisis. Environmental Justice, 9(5), 143–151.

[R60] Sadler RC, & Lafreniere D (2017). Racist housing practices as a precursor to uneven neighborhood change in a post-industrial city. Housing Studies, 32(2), 186–208.

[R61] Scott AJ (1998). Regions and the world economy: The coming shape of global production, competition, and political order. Oxford: Oxford University Press.

[R62] Squires GD, & Kubrin CE (2005). Privileged places: Race, uneven development and the geography of opportunity in urban America. Urban Studies, 42(1), 47–68.

[R63] St. Clair T, Wial H, & Wolman H. (2012). Chronically-distressed metropolitan area economies. Paper presented at the 42nd Urban Affairs Association Conference, Pittsburgh, PA.

[R64] Stansel D (2005). Local decentralization and local economic growth: A cross-sectional examination of US metropolitan areas. Journal of Urban Economics, 57(1), 55–72.

[R65] Storper M (1997). The regional world: Territorial development in a global economy. New York, NY: Guilford Press.

[R66] Sunley P (2000). Urban and regional growth. In Sheppard E & Barnes TJ (Eds.), A companion to economic geography (pp. 187–201). Hoboken, NJ: Blackwell.

[R67] Tighe JR, & Ganning JP (2015). The divergent city: Unequal and uneven development in St. Louis. Urban Geography, 36(5), 654–673.

[R68] University of Arkansas System. (n.d.). Campuses and units. University of Arkansas System. Retrieved from https://www.uasys.edu/campuses-units

[R69] van Agtmael A, & Bakker F (2016). The smartest places on earth: Why rustbelts are the emerging hotspots of global innovation. London: Hachette.

[R70] Weaver R (2017). Palliative planning in an American shrinking city—Some thoughts and preliminary policy analysis. Community Development, 48(3), 436–450.

[R71] Weiher G (1991). The fractured metropolis: Political fragmentation and metropolitan segregation. Albany, NY: SUNY Press.

[R72] Wheeler CH (2006). Urban decentralization and income inequality: Is sprawl associated with rising income segregation across neighborhoods? (Working Paper 2006–037B). St. Louis, MO: Federal Reserve Bank of St. Louis.

[R73] Wiewel W, & Schaffer K (2001). Learning to think as a region: Connecting suburban sprawl and city poverty. European Planning Studies, 9(5), 593–611.

[R74] Wolman H, Hill E, Blumenthal P, & Furdell K (2008). Understanding economically distressed cities. In McGahey RM & Vey JS (Eds.), Retooling for growth: Building a 21st century economy in America’s older industrial areas (pp. 151–178). Washington, DC: Brookings Institution Press.

